# Protective role of tissue-resident Tregs in a murine model of beryllium-induced disease

**DOI:** 10.1172/jci.insight.156098

**Published:** 2022-08-22

**Authors:** Shaikh M. Atif, Douglas G. Mack, Allison K. Martin, Andrew P. Fontenot

**Affiliations:** 1 Department of Medicine and; 2 Department of Immunology and Microbiology, University of Colorado Anschutz Medical Campus, Aurora, Colorado, USA.

**Keywords:** Pulmonology, Adaptive immunity, T cells, Th1 response

## Abstract

CD4^+^ T cells drive the immunopathogenesis of chronic beryllium disease (CBD), and their recruitment to the lung heralds the onset of granulomatous inflammation. We have shown that CD4^+^ Tregs control granuloma formation in an HLA-DP2 Tg model of CBD. In these mice, beryllium oxide (BeO) exposure resulted in the accumulation of 3 distinct CD4^+^ T cell subsets in the lung, with the majority of tissue-resident memory cells expressing FoxP3. The amount of Be regulated the number of total and antigen-specific CD4^+^ T cells and Tregs in the lungs of HLA-DP2 Tg mice. Depletion of Tregs increased the number of IFN-γ–producing CD4^+^ T cells and enhanced lung injury, while mice treated with IL-2/αIL-2 complexes had increased Tregs and reduced inflammation and Be-responsive T cells in the lung. BeO-experienced resident Tregs suppressed anti-CD3–induced proliferation of CD4^+^ T cells in a contact-dependent manner. CTLA-4 and ICOS blockade, as well as the addition of LPS to BeO-exposed mice, increased the effector T cell (Teff)/Treg ratio and enhanced lung injury. Collectively, these data show that the protective role of tissue-resident Tregs is dependent on quantity of Be exposure and is overcome by blocking immune regulatory molecules or additional environmental insults.

## Graphical abstract

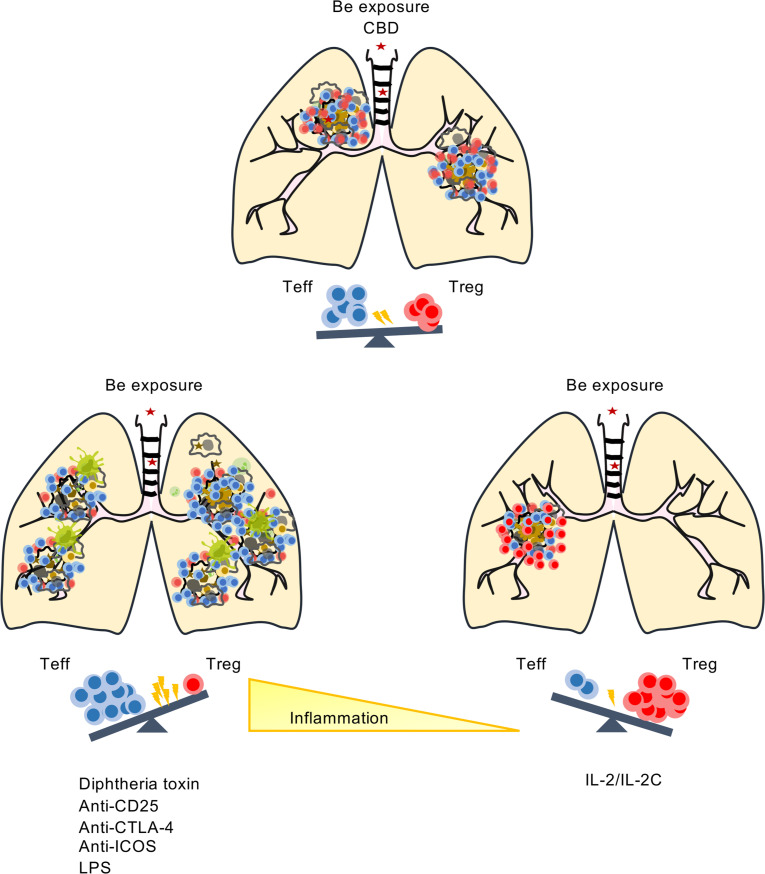

## Introduction

Chronic beryllium disease (CBD) is a metal-induced hypersensitivity that is characterized by activation of both the innate and adaptive immune systems (1–4). The development of a beryllium-specific (Be-specific) adaptive immune response is dependent on the presence of *HLA-DPB1* alleles containing a glutamic acid at position 69 (βGlu69) (4–7), and mutation of βGlu69 in HLA-DP molecules abrogates Be-induced CD4^+^ T cell activation (5, 8). Exposure to Be and other metals induces lung inflammation that drives the recruitment of leukocytes to the lung (9, 10). Early inflammation occurs through the release of host-derived danger-associated molecular patterns (DAMPs) released from dying alveolar macrophages (11) and injured epithelial cells. Besides DAMPs, exposure to sterile particles also induces the secretion of cytokines and chemokines that orchestrate the recruitment of inflammatory monocytes and activated T and B cells (12, 13). The innate induction of CCL3 and CCL4 allows the formation of Be-containing neoantigens that lead to the recruitment and expansion of HLA-DP2-CCL/Be–specific CD4^+^ Th1-polarized cells that promote the formation of cellular aggregates and/or granulomatous inflammation (12–14).

CD4^+^ T cells are an important component of the immune system involved in maintaining homeostasis and controlling infections (15). In CBD, pathogenic CD4^+^ Th1 cells are recruited to the lungs in response to Be exposure. In HLA-DP2 Tg mice, Be oxide (BeO) exposure induces the recruitment of neoantigen-reactive CD4^+^ T cells to the lung (13, 16, 17) and mononuclear infiltrates in a peribronchovascular distribution, replicating the major features of the human disease (3, 18). However, compared with CBD patients, the CD4^+^ T cell subset in the lungs of BeO-exposed HLA-DP2 Tg mice is composed of a much larger fraction of Tregs, comprising 25%–35% of lung CD4^+^ T cells (3). Naturally occurring Tregs are derived in the thymus and are characterized as CD4^+^ T cells expressing CD25 (IL-2 receptor α chain) and forkhead box protein 3 (FoxP3) (19, 20). Tregs play a key role in immunoregulation — in particular, suppressing inflammation to self- or foreign antigens in both lymphoid and nonlymphoid organs, thus controlling immune homeostasis (21, 22). Previously, we showed that BeO exposure resulted in the expansion of Tregs in the lungs of HLA-DP2 Tg mice and that CD25-mediated depletion of Tregs exacerbated disease pathology (3). Tregs are recruited to the nonlymphoid organs in response to various chemokines, including CXCL10, CCL19, and CCL21 (23, 24), and they suppress inflammation through multiple pathways, including the secretion of cytokines (e.g., IL-10 and/or TGF-β) and contact-dependent inhibition of T cell receptor signaling via expression of inducible T cell costimulator (ICOS) and/or CTLA-4, coreceptors involved in the development, homeostasis, and function of Tregs (25–27). However, the phenotype and mechanisms by which tissue-resident Tregs control BeO-induced inflammation are unknown.

In the present study, we utilized a well-characterized murine model of CBD to delineate the effects of BeO exposure on the phenotype and kinetics of tissue-resident CD4^+^ T cells and Tregs. Multiple BeO exposures increased the accumulation of tissue-resident (CD69^+^CD103^+^) Tregs that display enhanced expression of ICOS and CTLA-4 and tissue-resident effector (CD69^+^CD103^–^) FoxP3^–^ T cells. Additionally, multiple BeO exposures increased the number of CD4^+^ T cells specific for the HLA-DP2-CCL/Be complex as compared with a single dose of BeO. Depletion of Tregs in HLA-DP2-FoxP3-DTR Tg mice and anti-CD25 mAb–treated mice enhanced effector responses, while mice treated with IL-2/αIL-2 complexes suppressed the effector response by increasing the frequency of Tregs in the lungs of BeO-exposed HLA-DP2 Tg mice. The addition of LPS to BeO-exposed HLA-DP2 Tg mice increased the ratio of effector T cells (Teffs) to Tregs and enhanced lung inflammation and injury. Collectively, these data demonstrate the ability of BeO exposure to modulate the balance between Teffs and Tregs in the lung and the ability of Tregs to prevent excessive damage induced by pathogenic neoantigen-specific CD4^+^ T cells.

## Results

### BeO exposure induces accumulation of tissue-resident effector and memory cells in the lungs.

**Figure 1:**
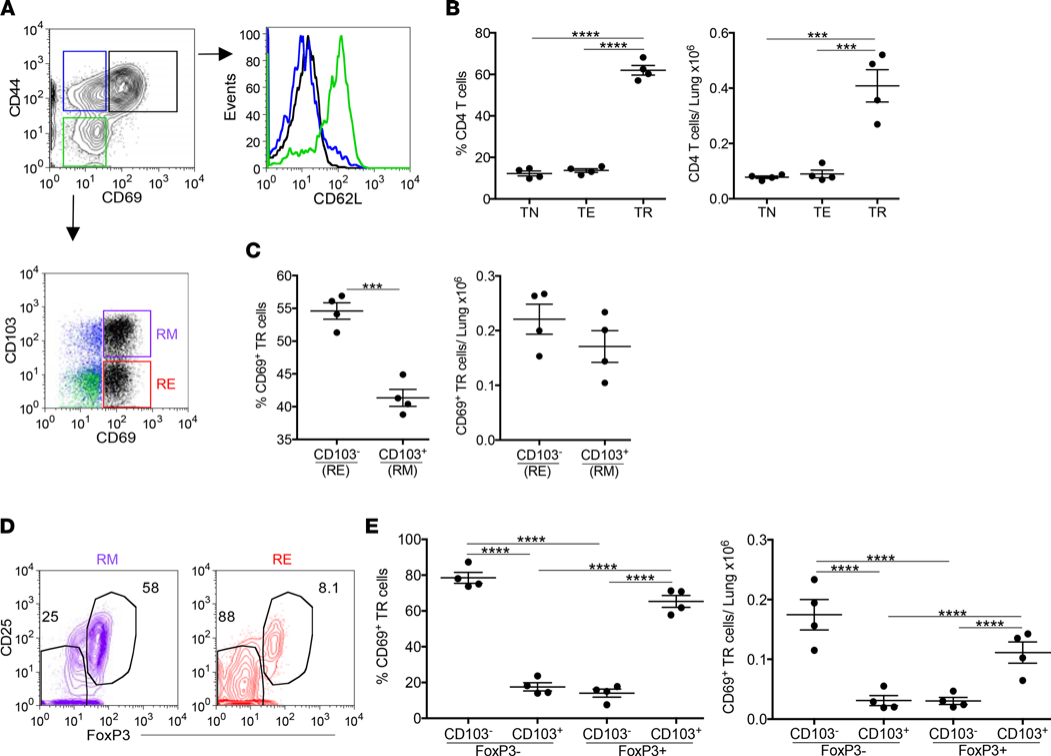
BeO exposure induces accumulation of FoxP3-expressing resident memory CD4^+^ T cells in the lung. HLA-DP2 Tg mice were exposed with 100 μg BeO, and lungs were harvested on day 21 and analyzed by flow cytometry. (**A**) Representative contour plot gated on lung CD4^+^ T cells show expression of CD44 and CD69. Three distinct CD4^+^ T cell subsets are enclosed in colored boxes: CD44^–^CD69^–^ naive T cells (TN, green), CD44^+^CD69^–^ Teffs (TE, blue), and CD44^+^CD69^+^ activated resident T cells (TR, black). Histogram shows CD62L expression on tissue-specific CD4^+^ naive T cells, Teffs, and activated resident T cells (same color coding as in **A**). Representative dot plot show CD103 and CD69 expression on CD4^+^ T cells. TR cells are broken down into resident memory (RM, purple box) and resident effector (RE, red box) T cells. (**B**) Frequency (left panel) and number (right panel) of CD4^+^ naive T cells, Teffs, and activated resident T cells in the lungs of BeO-exposed HLA-DP2 Tg mice. (**C**) Frequency (left panel) and number (right panel) of RE (CD103^–^CD69^+^) and RM (CD103^+^CD69^+^) CD4^+^ activated resident cells in the lungs of BeO-exposed HLA-DP2 Tg mice. (**D**) Representative contour plots showing expression of CD25 and FoxP3 staining on CD4^+^CD103^+^CD69^+^ RM T cells (purple) and CD4^+^CD103^–^CD69^+^ RE T cells (red). (**E**) Frequency (left panel) and number (right panel) of RM (CD103^+^CD69^+^) and RE (CD103^–^CD69^+^) CD4^+^ activated resident cells in the lungs of BeO-exposed HLA-DP2 Tg mice based on FoxP3 expression. Data (mean ± SEM) are representative of 3 independent experiments (3–5 mice per group). Student’s *t* test (**C**) and 1-way ANOVA (**B** and **E**) were used to test for differences. ***P < 0.001, **** *P* < 0.0001.

During CBD, Be exposure leads to accumulation of activated leukocytes in the airways and peribronchovascular region of the lungs (3, 28). Using HLA-DP2 Tg mice, we examined the phenotype of CD4^+^ T cells recruited to the lung at day 21 in response to BeO sensitization and boost (7 doses given on days 0, 1, 2, 14, 15, 18, and 19). CD4^+^ T cells in the lung were studied by excluding circulating cells using an i.v. injection of an anti-CD45 mAb prior to sacrifice (29), and our gating strategy to differentiate effector and resident memory (RM) CD4^+^ T cells based on CD103 and CD69 expression is shown in Supplemental Figure 1 (supplemental material available online with this article; https://doi.org/10.1172/jci.insight.156098DS1). In lung tissue, we observed 3 subsets of CD4^+^ T cells that were classified into naive T cells (CD44^lo^CD69^lo^, enclosed in green box), Teffs (CD44^hi^CD69^lo^, blue box), and activated resident T cells (CD44^hi^CD69^hi^, black box) (Figure 1A). Overall, the dominant CD4^+^ T cell subset in the lungs of BeO-exposed HLA-DP2 Tg mice was activated resident T cells (Figure 1, A and B). Next, we have examined CD62L expression on these T cell subsets and showed that only recently recruited naive T cells (green in Figure 1A) expressed CD62L. As shown in Figure 1A, naive T cells (green dots) lacked expression of CD69 and CD103, while a small population of Teffs expressed CD103 in the absence of CD69 (blue dots) (Figure 1A). Among activated CD69^+^ resident T cells, approximately 55% of cells lacked CD103 expression and were considered as resident effector (RE) T cells, while remaining cells showed increased expression of CD103 and were phenotypically confirmed to be RM T cells (Figure 1C). Cumulative percentage and number of CD103^–^ RE and CD103^+^ RM T cells in the lungs of BeO-exposed HLA-DP2 Tg mice are shown in Figure 1C. Next, we assessed FoxP3 expression among CD103^–^ RE and CD103^+^ RM T cells, and representative contour plots are shown in Figure 1D. While the majority of RM T cells expressed FoxP3, only a minority of RE T cells expressed FoxP3 (Figure 1, D and E). Collectively, these findings show that BeO exposure results in the accumulation of FoxP3-expressing RM Tregs and pathogenic RE T cells.

### Dose- and time-dependent increase in CD4^+^ T cells in the lungs of BeO-exposed HLA-DP2 Tg mice.

**Figure 2:**
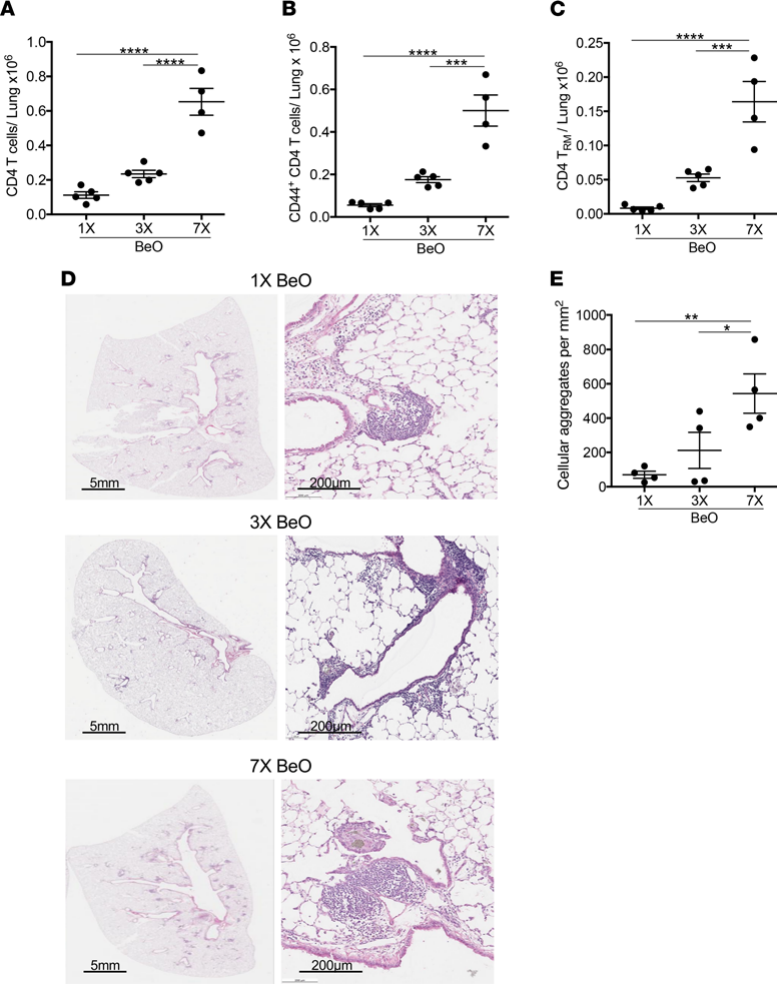
Beryllium exposure induces a dose-dependent increase in CD4^+^ T cell accumulation and mononuclear infiltrates in the lungs. HLA-DP2 Tg mice were exposed to 1 (1×), 3 (3×), or 7 (7×) doses of BeO (100 μg), and lungs were harvested on day 21. (**A**–**C**) Number of total CD4^+^ T cells (**A**), CD44^+^ Teffs (**B**), and CD103^+^CD69^+^ resident memory CD4^+^ T cells (**C**) in the lungs at day 21 were analyzed. (**D**) Representative H&E-stained lung sections of HLA-DP2 Tg mice treated with 1× (top), 3× (middle), and 7× (bottom) doses of BeO are shown at low and high magnification. (**E**) Quantification of cellular aggregates in the lungs of HLA-DP2 Tg mice exposed to 1 (1×), 3 (3×), or 7 (7×) doses of BeO. Data (mean ± SEM) are representative of 3 independent experiments (3–5 mice per group). One-way ANOVA was used to test for differences. *P < 0.05, **P < 0.01, ***P < 0.001, ****P < 0.0001.

To examine how the dose of BeO modulates CD4^+^ T cell recruitment to the lungs, we compared HLA-DP2 Tg mice that received either a single dose of BeO on day 0, BeO sensitization (3 doses of BeO given on days 0, 1, and 2), or BeO sensitization/boost and euthanization on day 21. HLA-DP2 Tg mice that received BeO sensitization/boost showed significantly increased numbers of total CD4^+^ T cells (Figure 2A), CD44^+^CD4^+^ Teffs (Figure 2B), and RM CD4^+^ T cells (Figure 2C) in the lungs compared with mice exposed to either 1 or 3 doses of BeO. Similarly, on day 12, BeO-sensitized HLA-DP2 Tg mice had an increased number of CD4^+^ T cells and memory CD4^+^ T cells (both effector and resident) in the lungs compared with mice that received 1 BeO exposure (Supplemental Figure 2, A–C). Histologically, HLA-DP2 Tg mice that received BeO sensitization/boost had increased peribronchovascular mononuclear infiltrates compared with mice that received either a single BeO dose or sensitization (Figure 2D), as well as a significant increase in cellular aggregates on quantitative analysis (Figure 2E). Conversely, no differences were seen in the number of cellular aggregates at day 12 after 1 or 3 BeO exposures (Supplemental Figure 2, D and E).

### Lung-resident CD4^+^ Tregs upregulate ICOS and CTLA-4 in BeO-exposed HLA-DP2 Tg mice.

**Figure 3:**
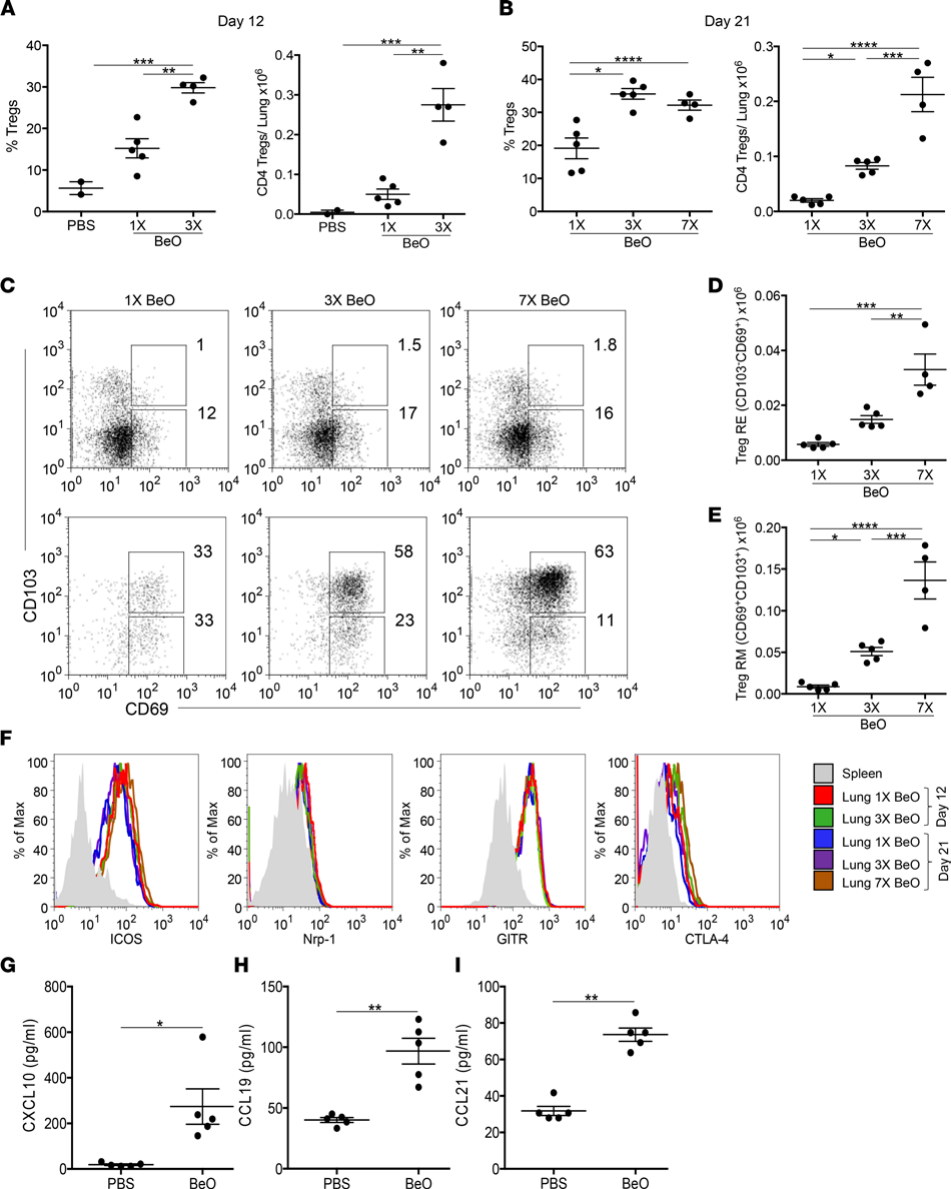
BeO exposure induces a dose-dependent increase in tissue-resident Tregs in the lung. Frequency (left panel) and number (right panel) of CD4^+^CD25^+^FoxP3^+^ T cells in the lungs of HLA-DP2 Tg mice sensitized with 1 (1×) or 3 (3×) doses of BeO and harvested at day 12 (**A**) or sensitized with 1 (1×), 3 (3×), or 7 (7×, sensitization/boost) BeO exposures and examined at day 21 (**B**). (**C**) Representative dot plots show CD103 and CD69 expression on CD4^+^CD25^+^FoxP3^+^ Tregs derived from the spleen (top panels) and lung (bottom panels) of mice exposed to BeO on 1 (1×), 3 (3×), or 7 (7×) occasions and examined at day 21. (**D** and **E**) Number of resident effector (RE, CD103^–^CD69^+^) and resident memory (RM, CD103^+^CD69^+^) T cells among tissue-resident Tregs in HLA-DP2 Tg mice exposed to 1, 3, and 7 doses of BeO. (**F**) Representative histograms show expression of ICOS, Nrp-1, GITR, and CTLA-4 on tissue-specific CD25^+^FoxP3^+^CD4^+^ Tregs in mice exposed to 1 (1×, red) or 3 (3×, green) doses of BeO and analyzed at day 12 or exposed to 1 (1×, blue), 3 (3×, purple), or 7 (7×, brown) doses of BeO and analyzed at day 21. (**G**–**I**) T cell homeostatic chemokines CXCL-10 (**G**), CCL19 (**H**), and CCL21 (**I**) were measured in the lung tissue lysates by ELISA. Data (mean ± SEM) are representative of 3 individual experiments (2–5 mice per group). Significance was determined by 1-way ANOVA (**A**, **B**, **D**, and **E**) and Mann-Whitney *U* test (**G**–**I**). *P < 0.05, **P < 0.01, ***P < 0.001, ****P < 0.0001.

We have previously shown that BeO exposure resulted in the accumulation of FoxP3^+^ Tregs in the lungs (3). However, whether these cells were within the lung circulation or were true resident cells was not examined. We evaluated the frequency and number of tissue-resident CD25^+^FoxP3^+^ Tregs in HLA-DP2 Tg mice that received either 1 BeO exposure, BeO sensitization, or BeO sensitization/boost. At day 12, HLA-DP2 Tg mice sensitized to BeO showed an increased frequency and number of Tregs compared with mice that received 1 exposure of BeO or PBS (Figure 3A). Similarly, BeO-sensitized and BeO-sensitized/boosted mice had an increased frequency and number of FoxP3^+^ Tregs in the lungs compared with mice exposed to a single dose of BeO at day 21 (Figure 3B). Importantly, lung-resident Tregs expressed high levels of CD69 and CD103 compared with splenic Tregs (Figure 3C), and the number of CD69^+^CD103^–^ RE and CD69^+^CD103^+^ RM CD4^+^ Tregs significantly increased with repeated BeO exposure (Figure 3, D and E).

Next, we examined the phenotype of lung-resident Tregs compared with splenic Tregs using well-established markers. On day 12 or 21 after sensitization or sensitization/boost with BeO, lung-resident Tregs expressed increased levels of ICOS, glucocorticoid-induced tumor necrosis factor receptor-related protein (GITR), and cytotoxic induced lymphocyte antigen 4 (CTLA-4) compared with splenic Tregs, while no or little difference was noted in the expression of neuropilin-1 (Nrp-1) (Figure 3F). A dose-dependent increase in the expression of CTLA-4 and ICOS on Tregs in BeO-sensitized or BeO-sensitized/boosted HLA-DP2 Tg mice was observed compared with mice exposed to PBS or 1 dose of BeO (Supplemental Figure 3). Furthermore, at day 21, BeO-sensitized/boosted mice showed significantly increased levels of CXCL10, CCL19, and CCL21, chemokines involved in the recruitment of CD4^+^ T cells to the lung (Figure 3, G–I) (23, 24). Overall, these data demonstrate a significant increase in Tregs in the lungs with BeO sensitization/boost and identified the expression of molecules that are selectively upregulated on lung-resident Tregs compared with peripheral Tregs.

### BeO sensitization and boost of HLA-DP2 Tg mice induces epitope-specific Tregs.

**Figure 4:**
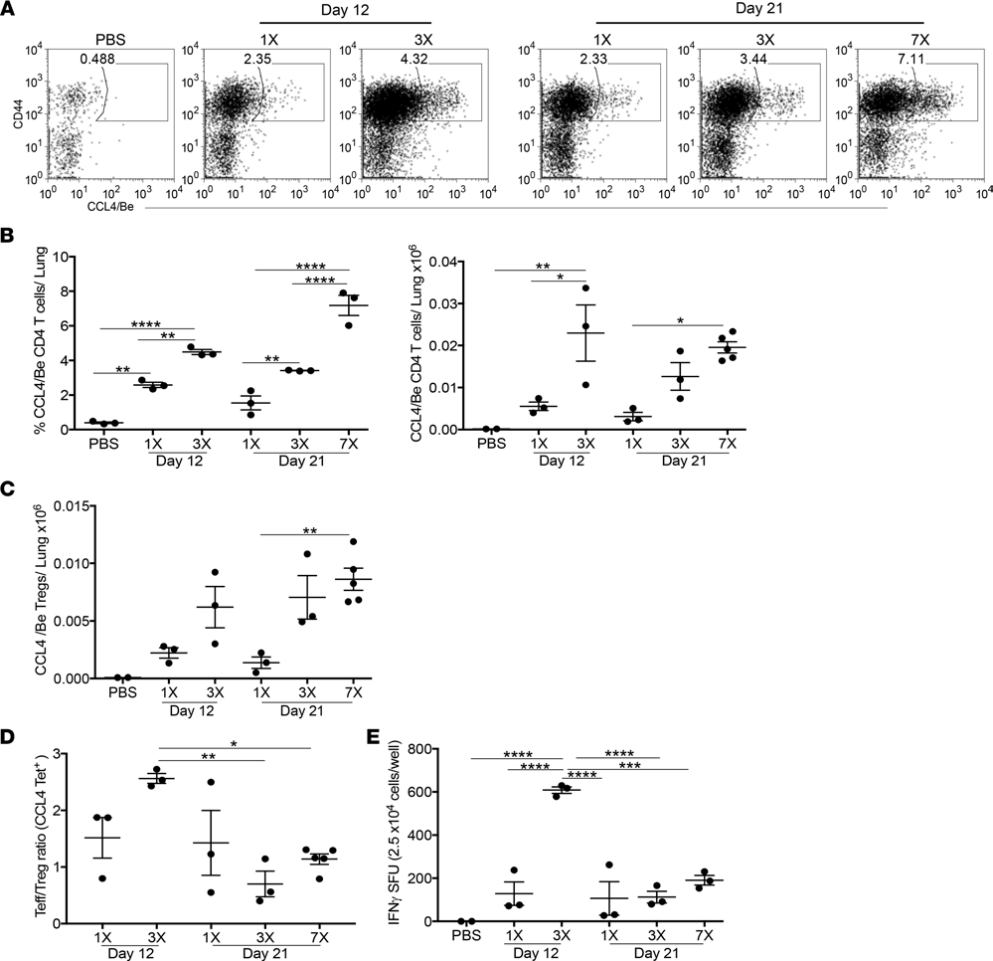
Increased frequency of antigen-specific Tregs correlates with reduced IFN-γ production. (**A**) Representative dot plots showing the frequency of CD4^+^ T cells specific for the HLA-DP2-CCL4/Be complex in the lungs of HLA-DP2 Tg mice sensitized with 1 (1×) or 3 (3×) doses of BeO and examined at day 12 or treated with 1 (1×), 3 (3×), or 7 (7×) doses of BeO and examined at day 21. (**B**) Frequency (left) and number (right) of HLA-DP2-CCL4/Be–specific CD4^+^ T cells in the lungs of HLA-DP2 Tg mice exposed to a differing number of BeO doses and analyzed at day 12 or day 21. (**C**) Number of HLA-DP2-CCL4/Be–specific CD4^+^ Tregs in the lungs of HLA-DP2 Tg mice. (**D**) Ratio of HLA-DP2-CCL4/Be–specific CD44^+^ Teff/CD25^+^FoxP3^+^ Tregs in the lungs of HLA-DP2 Tg mice exposed with single or multiple doses of BeO and analyzed at day 12 or 21. (**E**) IFN-γ secretion by CD4^+^ T cells purified from the lungs of HLA-DP2 Tg mice exposed to a single or multiple doses of BeO, harvested at day 12 or 21, and stimulated with BeSO_4_ (100 μM) in the presence of irradiated naive splenocytes for 24 hours. Data (mean ± SEM) are representative of 3 independent experiments (3–5 mice per group). Significance was determined by 1-way ANOVA. *P < 0.05, **P < 0.01, ***P < 0.001, ****P < 0.0001.

The accumulation of IFN-γ–producing Be-specific CD4^+^ T cells in the BAL is a hallmark of Be-induced disease (14, 30). However, compared with CBD subjects where Tregs are deficient in the BAL (31), we identified an expansion of CD4^+^ Tregs in the lungs of BeO-exposed HLA-DP2 Tg mice (Figure 3). Recently, we showed that CD4^+^ T cells derived from the lungs of HLA-DP2–expressing CBD subjects and mice recognize a T cell epitope composed of HLA-DP2-CCL/Be (13). Here, we examined the accumulation of epitope-specific CD4^+^ T cells, as well as the total Be-responsive T cell population in the lungs after Be sensitization at day 12 and after Be sensitization or Be sensitization/boost at day 21. Mice exposed to multiple doses of BeO showed an increased frequency and number of HLA-DP2-CCL4/Be–specific CD4^+^ T cells compared with a single dose of BeO (Figure 4, A and B). Similarly, an increased number of HLA-DP2-CCL4/Be–specific CD4^+^ Tregs was seen in the lungs of mice after Be sensitization and Be sensitization/boost compared with mice exposed to a single BeO inhalation (Figure 4C).

To investigate the effects of increased antigen-specific Tregs on the development of the Be-specific adaptive immune response, we examined the ratio of epitope-specific CD4^+^ Teffs to Tregs using tetramer staining (Figure 4D). Be-sensitized mice had a significantly increased Teff/Treg ratio of epitope-specific CD4^+^ T cells at day 12 compared with Be-sensitized/boosted mice analyzed at day 21 (Figure 4D). The increased Teff/Treg ratio after BeO sensitization was associated with a significantly increased number of IFN-γ–secreting cells in the lungs at day 12 compared with Be-sensitized/boosted mice analyzed at day 21 (Figure 4E). Importantly, a greater-than 2-fold reduction in the Teff/Treg ratio of epitope-specific CD4^+^ T cells and a ~3-fold reduction in the total number of IFN-γ–secreting cells was seen in HLA-DP2 Tg mice that received BeO sensitization/boost compared with sensitization alone (Figure 4, D and E). Collectively, these data show that, despite similar numbers of CD4^+^ T cells in the lungs after BeO sensitization and BeO sensitization/boost, the composition of the lung-resident CD4^+^ T cell pool shifts away from IFN-γ–expressing Teffs toward a Treg subset.

### Modulation of CD25-expressing Tregs alters lung injury in BeO-exposed mice.

**Figure 5:**
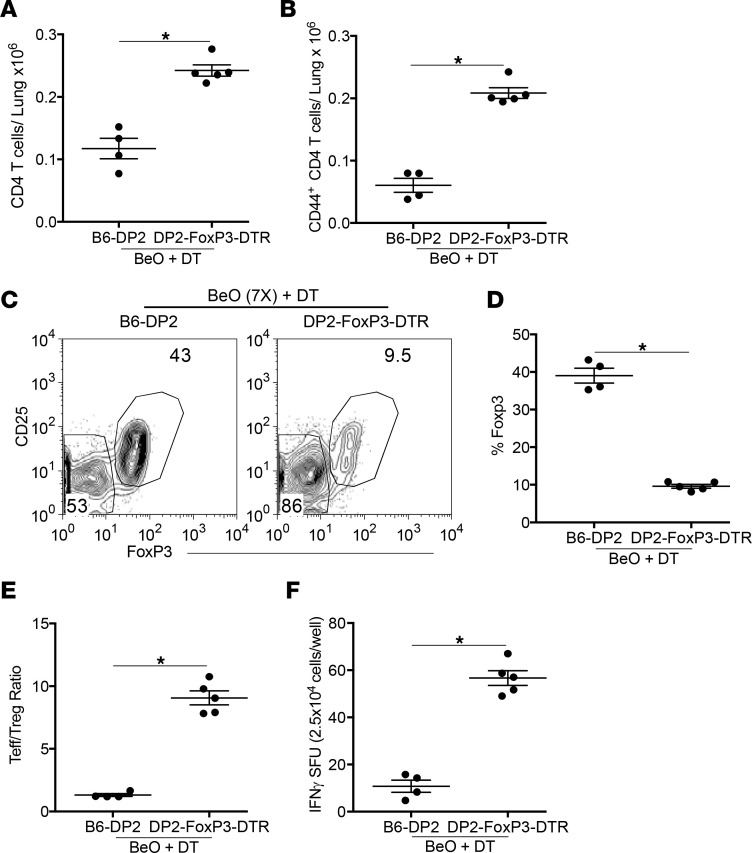
Depletion of Tregs in HLA-DP2-FoxP3-DTR Tg mice alters the Teff/Treg ratio and the number of IFN-γ–secreting T cells in the lung. (**A** and **B**) Number of CD4^+^ T cells (**A**) and effector CD44^+^CD4^+^ T cells (**B**) in the lungs on day 21 in HLA-DP2 Tg and HLA-DP2-FoxP3-DTR Tg mice sensitized and boosted with 7 (7×) doses of BeO on days 0, 1, 2, 14, 15, 18, and 19, and treated with 1 μg of diphtheria toxin (DT) on days 18 and 19 is shown. (**C** and **D**) Representative contour plots (**C**) and the cumulative frequency (**D**) of CD25^+^FoxP3^+^ Tregs on day 21 in the lungs of HLA-DP2 Tg (left contour plot) and HLA-DP2-FoxP3-DTR Tg mice (right contour plot) after DT treatment. (**E**) Ratio of CD44^+^ Teffs/CD25^+^FoxP3^+^ Tregs in the lungs of HLA-DP2 Tg and HLA-DP2-FoxP3-DTR Tg mice exposed with multiple doses of BeO and analyzed at day 21. (**F**) IFN-γ secretion by CD4^+^ T cells purified from the lungs of HLA-DP2 Tg and HLA-DP2-FoxP3-DTR Tg mice exposed to multiple doses of BeO, harvested at day 21, and stimulated with BeSO_4_ (100 μM) in the presence of irradiated naive splenocytes for 24 hours. Data (mean ± SEM) are representative of 3 independent experiments (4–5 mice per group). Significance was determined by Mann-Whitney *U* test. *P < 0.05.

To understand the role of tissue-resident Tregs in controlling lung inflammation or damage caused by IFN-γ–producing CD4^+^ T cells during CBD, we used genetically engineered FoxP3-DTR Tg mice expressing the HLA-DP2 Tg (denoted as HLA-DP2-FoxP3-DTR Tg mice). HLA-DP2 Tg and HLA-DP2-FoxP3-DTR Tg mice were exposed to BeO using our standard sensitization/boost protocol and treated with diphtheria toxin (DT) on days 18 and 19. Total CD4^+^ T cells, CD44^+^ Teffs, and FoxP3^+^ Tregs were analyzed in the lungs on day 21. HLA-DP2-FoxP3-DTR Tg mice showed increased numbers of total CD4^+^ T cells and tissue-RE CD44^+^ T cells on day 21 (Figure 5, A and B). Contour plots show the reduced frequency of CD25^+^FoxP3^+^ Tregs in the lungs of DT-exposed HLA-DP2-FoxP3-DTR Tg mice (Figure 5C), while the cumulative frequency of CD4^+^ Tregs was also significantly reduced compared with the DT-exposed HLA-DP2 Tg mice (Figure 5D). Next, we examined the loss of Tregs on the ratio of Teffs and Tregs. Mice depleted of Tregs showed an increased Teff/Treg ratio, suggesting an increased effector Th1 response (Figure 5E). To corroborate the effect of Treg depletion on the increased Th1 response, we measured IFN-γ–producing CD4^+^ T cells in the lungs of BeO-exposed, DT-treated HLA-DP2 Tg, and HLA-DP2-FoxP3-DTR Tg mice (Figure 5F). Mice with reduced Tregs in the lungs showed a significant increase in the number of IFN-γ–producing CD4^+^ T cells compared with Treg-sufficient HLA-DP2 Tg mice, suggesting a protective role of Tregs in this murine model of Be-induced disease.

To assess the protective role of Tregs in the acute phase of BeO exposure, we modulated the number of Tregs using either an anti-CD25 mAb (PC61) to deplete Tregs or IL-2/αIL-2 complexes to increase Tregs. To confirm the functionality of PC61 and IL-2/αIL-2 complexes, we examined the frequency of Tregs in the blood at day 8. Mice treated with PC61 had a significantly reduced frequency of Tregs in blood as compared with isotype-treated mice (Supplemental Figure 4). Conversely, treatment with IL-2/αIL-2 complexes increased the frequency of Tregs in blood more than 2-fold compared with the isotype-treated mice (Supplemental Figure 4).

**Figure 6:**
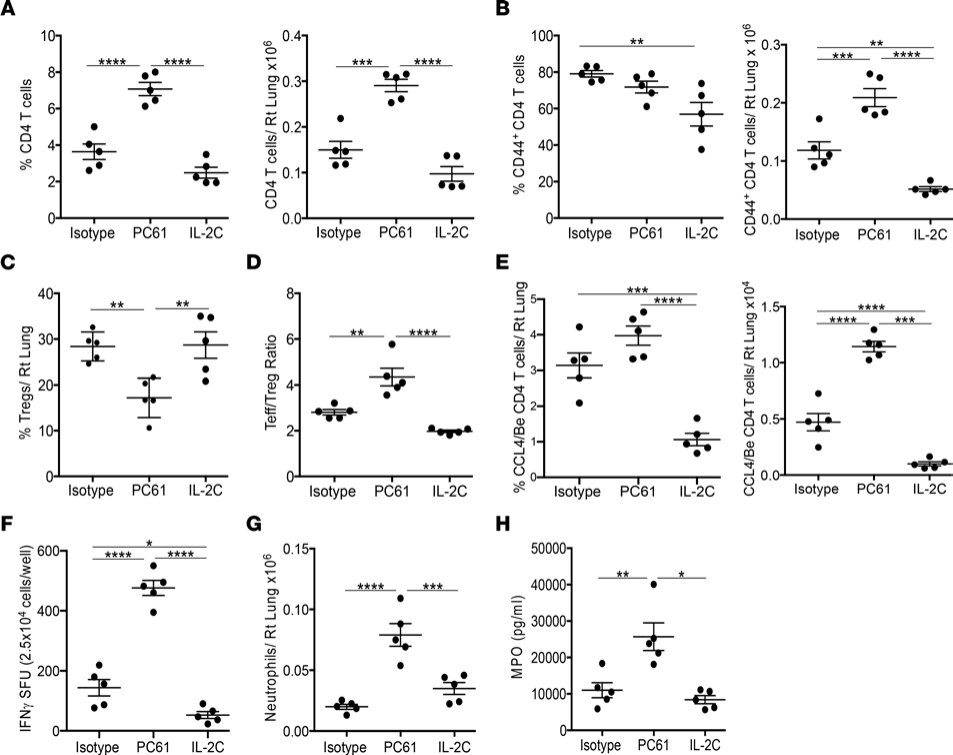
Loss or gain of Tregs modulates the Be-specific adaptive immune response. HLA-DP2 Tg mice were exposed to 3 doses of BeO and treated with either isotype control antibody, anti-CD25 mAb (PC61) at days –2 and 7, or IL-2/αIL-2 complexes (IL-2C, IL-2 combined with anti-IL-2 [JES6-1]) at days –2, 0, 2, and 7 and analyzed at day 12. (**A** and **B**) At day 12, the frequency (left panel) and number (right panel) of total CD4^+^ T cells (**A**) and CD4^+^CD44^+^ Teffs (**B**) in the lungs are shown. (**C**) Percentage of CD4^+^FoxP3^+^ Tregs in the lungs of BeO-exposed HLA-DP2 Tg mice. (**D**) Ratio of CD44^+^ Teffs to Tregs in the lungs of BeO-exposed HLA-DP2 Tg mice. (**E**) Frequency (left panel) and number (right panel) of HLA-DP2-CCL4/Be–specific CD4^+^ CD44^+^ T cells in the lungs of BeO-sensitized HLA-DP2 Tg mice. (**F**) IFN-γ secretion by CD4^+^ T cells purified from the lungs of HLA-DP2 Tg mice and stimulated with BeSO_4_ (100 μM) in the presence of irradiated naive splenocytes. (**G**) Number of Ly6G^+^ neutrophils in the lungs of HLA-DP2 Tg mice. (**H**) Myeloperoxidase (MPO) in the BALF of HLA-DP2 Tg mice. Data (shown as mean ± SEM) are representative of 3 individual experiments (5 mice per group). Significance was determined by 1-way ANOVA. *P < 0.05, **P < 0.01, ***P < 0.001, ****P < 0.0001.

BeO-sensitized mice (3 doses of BeO at days 0, 1, and 2) that were treated with PC61 showed an overall increase in the frequency and number of CD4^+^ T cells in the lung compared with the isotype-treated control or mice treated with IL-2/αIL-2 complexes (Figure 6A). Next, we examined the frequency and number of CD4^+^ Teffs in the lung and found a significantly increased number of Teffs in mice depleted of Tregs, while the number of Teffs in the lung was greatly reduced in the mice treated with IL-2/αIL-2 complexes compared with both isotype- and PC61-treated mice (Figure 6B). Mice treated with PC61 had a significantly decreased frequency of FoxP3^+^ Tregs compared with mice that received isotype or IL-2/αIL-2 complexes (Figure 6C). Surprisingly, the frequency of Tregs in the lungs of IL-2/αIL-2 complex–treated mice was similar to that seen in isotype-treated, BeO-exposed HLA-DP2 Tg mice (Figure 6C). Although the number of Tregs in the lungs of PC61-treated mice was unchanged compared with isotype-treated mice (data not shown), the ratio of Teff (total CD44^hi^ cells)/Tregs was significantly increased in PC61-treated mice compared with mice treated with either isotype control or IL-2/αIL-2 complexes (Figure 6D). HLA-DP2 Tg mice depleted of Tregs also showed an increase in the frequency and number of epitope-specific CD4^+^ T cells over isotype-treated controls or IL-2/αIL-2 complex–treated mice (Figure 6E). The increased Teff/Treg ratio in mice depleted of Tregs corresponded to an increased number of IFN-γ–producing CD4^+^ T cells in the lung (Figure 6F), while treatment with IL-2/αIL-2 complexes resulted in a significant reduction in the number of IFN-γ–producing cells in the lung in response to BeSO_4_ stimulation (Figure 6F). Next, we assessed lung injury in the treatment groups by examining neutrophil accumulation in the lungs and myeloperoxidase (MPO) in the BALF. Mice depleted of Tregs showed enhanced accumulation of neutrophils in the lungs (Figure 6G) and an increase in MPO levels in the BALF (Figure 6H), confirming the suppressive role of Tregs during Be-induced lung inflammation.

### BeO-exposed Tregs suppress T cell proliferation in a contact-dependent manner.

**Figure 7:**
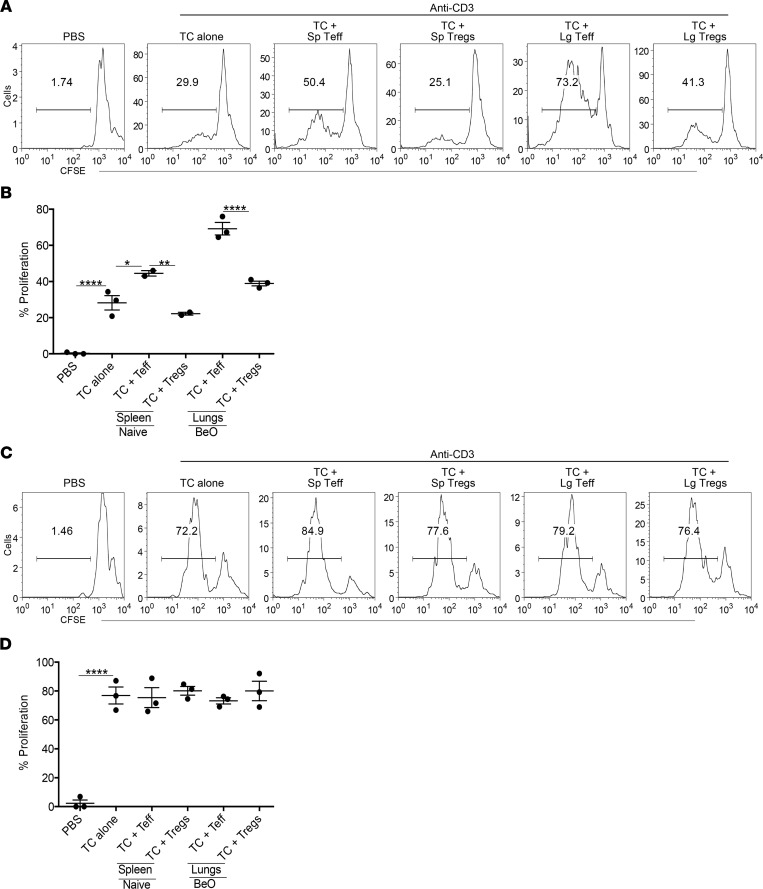
Beryllium-experienced Tregs suppress in vitro proliferation of anti-CD3 activated CD4^+^ T cells in a contact-dependent fashion. (**A** and **B**) Representative histograms and cumulative frequency data show proliferation of CFSE-labeled naive CD4^+^ T cells (TC) stimulated with anti-CD3 (2 μg/mL) and cultured in vitro in a 1:1 ratio with lung-resident (Lg-resident) effector (CD44^+^CD25^–^) or regulatory (CD25^+^FoxP3^+^) CD4^+^ T cells obtained at day 21 from BeO-sensitized/boosted HLA-DP2 Tg mice. Peripheral T effectors (CD44^+^CD25^–^) and Tregs (CD25^+^FoxP3^+^) sorted from the spleen (Sp) of naive mice were used as control cells. (**C** and **D**) Transwell assay was performed using CFSE-labeled T cells added to the bottom of 96-well transwell plates coated with anti-CD3 mAb (2 μg/mL). CD25^+^FoxP3^+^ Tregs and CD44^+^CD25^–^ cells sorted from the lungs of BeO-exposed HLA-DP2 Tg mice and peripheral T effectors (CD44^+^CD25^–^) and Tregs (CD25^+^FoxP3^+^) sorted from naive HLA-DP2 Tg mouse spleen were added at the top of the transwell. Representative histograms (**C**) and cumulative frequency data (**D**) shows T cell proliferation (loss of CFSE) at day 5. Data (mean ± SEM) are representative of 3 individual experiments. Significance was determined by 1-way ANOVA. *P < 0.05, **P < 0.01, ****P < 0.0001.

Tregs suppress inflammation acting either directly on CD4^+^ T cells or indirectly by maintaining the population of IL-10–producing macrophages in the lungs (32). To examine the suppressive ability of BeO-exposed Tregs, we performed in vitro CD4^+^ T cell suppression assays. Naive CD4^+^ conventional T cells (Tconv cells) were bead purified from the spleen of an untreated HLA-DP2 Tg mouse and labeled with 1 μM CFSE. CFSE-labeled Tconv cells were cocultured in 96-well plates in the presence of anti-CD3 (2 μg/mL) and sorted Teffs (CD44^+^CD25^–^) or Tregs (CD25^+^) from purified CD4^+^ T cells enriched from the lungs of BeO-exposed mice or spleens of PBS-treated mice at day 21 in a 1:1 ratio for 5 days. Proliferation of CD4^+^ Tconv cells was almost 50% inhibited in wells cocultured with lung Tregs (Figure 7A). Similar suppressive capacity of lung-derived Tregs was seen when CFSE-labeled Tconv cells were stimulated with 1 μg/mL of anti-CD3 (Supplemental Figure 5). Suppression of Tconv cell proliferation was also observed in wells cocultured with splenic Tregs from PBS-treated mice (Figure 7A) or Tregs purified from the spleens of BeO-exposed mice (data not shown). As expected, increased proliferation of Tconv cells was seen when Tconv cells were cultured with sorted Teffs (CD44^+^CD25^–^) from either the lung or spleen from BeO-exposed mice (Figure 7A).

To determine the mechanisms employed by Tregs to mediate suppression, we used a transwell assay and stimulated cells with plate-bound anti-CD3 (2 μg/mL). Tconv cells were added to the bottom of the plates, and Teff or Tregs isolated from the lungs of BeO-exposed mice and spleen of PBS-treated mice were added to the upper transwell section (Figure 7B). Loss of CFSE was measured by flow cytometry at day 5. Proliferation of Tconv cells was not inhibited in the transwell assay (Figure 7, C and D), suggesting that Treg-mediated suppression was contact dependent.

### Blocking of ICOS and CTLA-4 abrogate Treg-mediated suppression in BeO-exposed mice.

**Figure 8:**
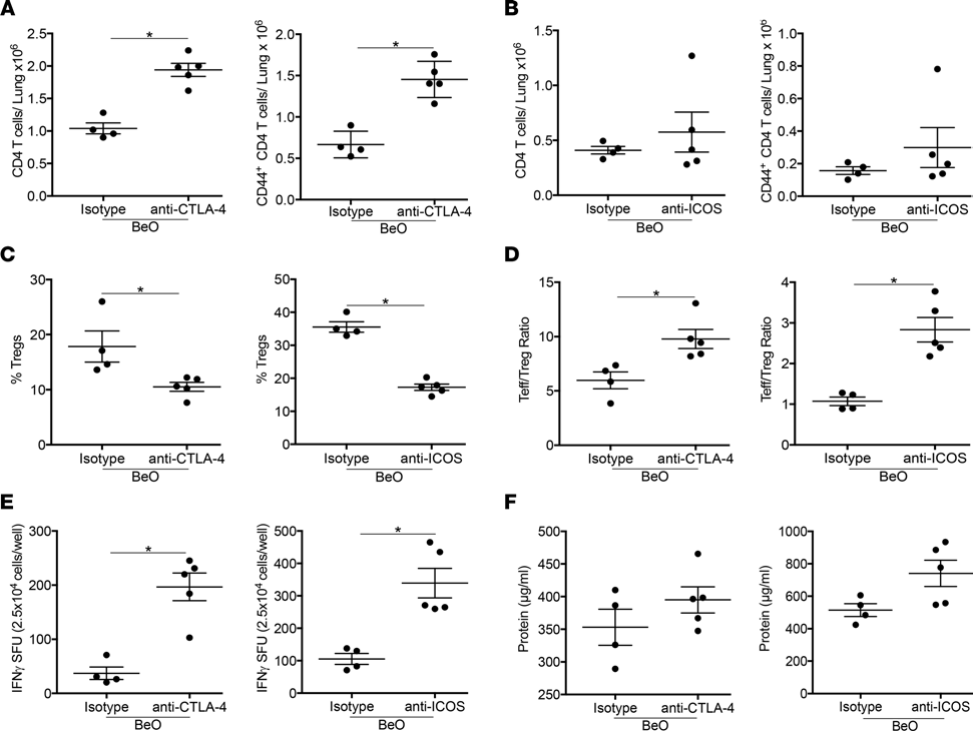
Tregs suppress BeO-induced inflammation through CTLA-4 and ICOS. (**A** and **B**) Plots show the number of CD4^+^ T cells (left panel) and CD44^+^ T effector cells (right panel) in the lungs of BeO-sensitized HLA-DP2 Tg mice on day 12 treated i.p. with either an isotype control antibody and anti–CTLA-4 (**A**) or anti-ICOS (**B**) blocking antibodies at day –1. (**C**) Percentage of FoxP3^+^ Tregs in the lungs of isotype and anti–CTLA-4 (left) or anti-ICOS (right) blocking antibody–treated, BeO-exposed HLA-DP2 Tg mice on day 12. (**D**) At day 12, Teff/Treg ratio was calculated by dividing the total number of tissue-specific Teffs (CD44^+^) by total Tregs (CD25^+^FoxP3^+^) in anti–CTLA-4 (left) and anti-ICOS (right) antibody–treated HLA-DP2 Tg mice. (**E**) IFN-γ secretion by CD4^+^ T cells purified from the lungs of BeO-exposed and anti–CTLA-4 (left) or anti-ICOS (right) blocking antibody–treated HLA-DP2 Tg mice and stimulated with BeSO_4_ (100 μM) in the presence of irradiated naive splenocytes. (**F**) Protein in the BALF of mice exposed with BeO (3×) and treated with anti–CTLA-4 (left) or anti-ICOS (right) blocking antibody was examined by ELISA. Data (mean ± SEM) are representative of 2 individual experiments (4–5 mice per group). Significance was determined by Mann-Whitney *U* test. *P < 0.05.

CTLA-4 and ICOS mediate the suppressive function of Tregs by inhibiting the maturation of antigen presenting cells (33). To investigate the role of CTLA-4 and ICOS in Treg-mediated suppression in our model of Be-induced disease, we used blocking antibodies directed against CTLA-4 or ICOS. HLA-DP2 Tg mice were either treated with isotype or blocking antibodies on day –1 and sensitized with BeO on days 0, 1, and 2. Mice treated with anti–CTLA-4 blocking antibody showed an increased number of CD4^+^ T cells and CD44^+^ T effector cells in the lungs on day 12 compared with isotype-treated control mice (Figure 8A), while no significant differences were observed in the anti-ICOS antibody–treated group compared with the control group (Figure 8B). The frequency of Tregs in the lung was significantly reduced in mice treated with CTLA-4– and ICOS-blocking antibodies compared with the isotype control–treated mice at day 12 (Figure 8C). Next, we examined the ratio of Teffs to Tregs and the accumulation of IFN-γ–producing CD4^+^ T cells in the lungs of BeO-exposed HLA-DP2 Tg mice treated CTLA-4– and ICOS-blocking antibodies. Mice treated with anti–CTLA-4 or anti-ICOS antibodies showed an elevated Teff/Treg ratio (Figure 8D) and an increased number of IFN-γ–producing CD4^+^ T cells compared with the control group (Figure 8E). While a slightly higher protein level was seen in the BALF of mice treated with anti-ICOS or anti–CTLA-4 antibodies compared with the isotype control–treated mice, the differences did not reach statistical significance (Figure 8F). Overall, these data suggest a role for CTLA-4 and ICOS in Treg-mediated suppression in this murine model of CBD.

### LPS modulates Be-induced tolerance in the lungs.

**Figure 9:**
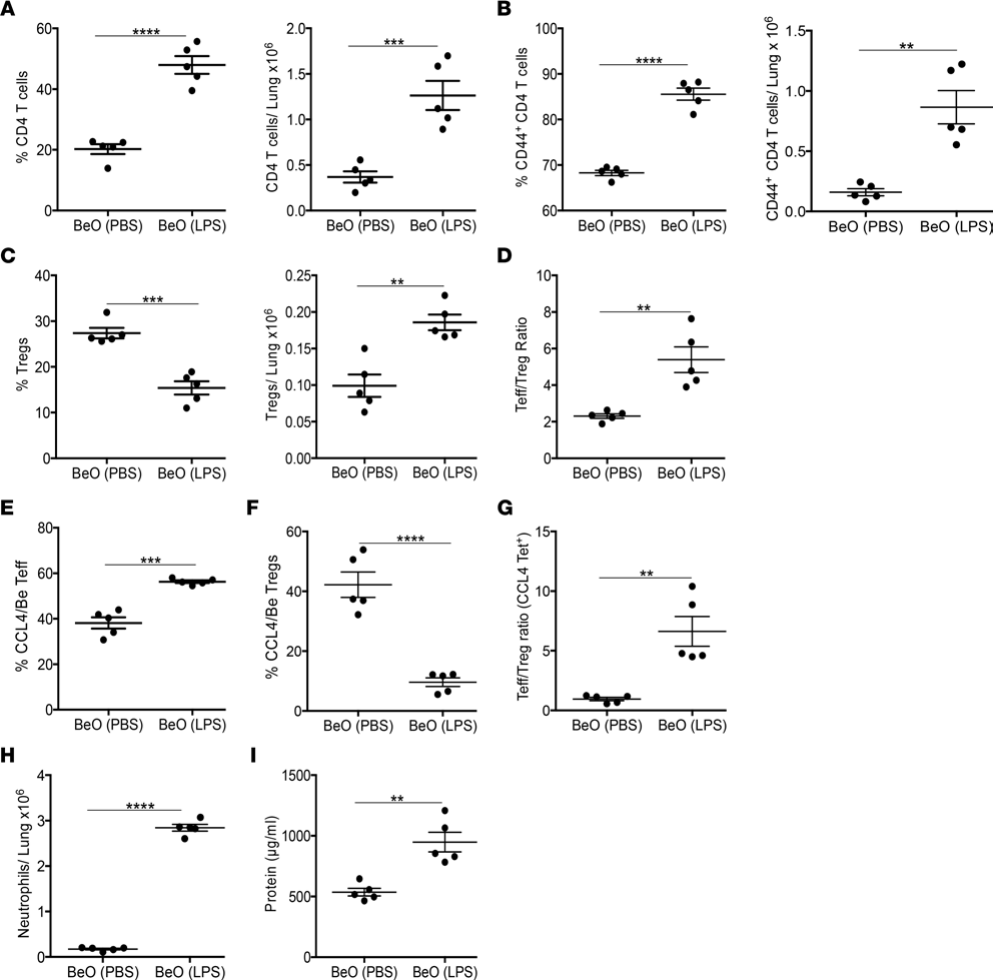
LPS enhances Be-induced lung injury by altering the Teff/Treg ratio. (**A**–**C**) Frequency (left panel) and number (right panel) of CD4^+^ T cells (**A**), CD4^+^CD44^+^ T effector cells (Teff) (**B**), and CD4^+^CD25^+^FoxP3^+^ Tregs (**C**) were examined at day 21 in HLA-DP2 Tg mice sensitized/boosted with BeO (100 μg) with or without administration of LPS (10 μg) on day 14. (**D**) At day 21, the Teff/Treg ratio was calculated by dividing the total number of tissue specific Teff cells (CD44^+^) by total Tregs (CD25^+^FoxP3^+^). (**E** and **F**) Frequency of HLA-DP2-CCL4/Be tetramer-binding CD4^+^ Teffs (**E**) and Tregs (**F**) in the lungs of BeO-exposed HLA-DP2 Tg mice with or without LPS treatment. (**G**) Ratio of the total number of HLA-DP-–CCL4/Be tetramer-binding CD4^+^ Teff to Tregs at day 21. (**H**) Number of Ly6G^+^ neutrophils in the lungs of BeO-exposed HLA-DP2 Tg mice in the presence or absence of LPS treatment. (**I**) Protein in the BALF of BeO-exposed HLA-DP2 Tg mice in the presence or absence of LPS. Data are shown as mean ± SEM. Data are representative of 2 independent experiments (5 mice per group). Significance was determined by Student’s *t* test. *P < 0.05, **P < 0.01, ***P < 0.001, ****P < 0.0001.

To assess the effects of sterile/pathogenic inflammation on the Be-induced CD4^+^ Treg response, we exposed HLA-DP2 Tg mice to a single dose of LPS at day 14 during BeO sensitization/boost. BeO-sensitized/boosted mice that received LPS showed an increased frequency and number of total CD4^+^ T cells (Figure 9A) and Teffs (Figure 9B) in the lung compared with mice treated with BeO alone. However, LPS exposure reduced the frequency of CD4^+^ Tregs (Figure 9C, left panel), while the number of Tregs in the lung was increased compared with BeO-exposed mice (Figure 9C, right panel). Since LPS induced robust Teff responses accompanied by increased IFN-γ–secreting CD4^+^ T cells in the lung (13), we measured the ratio of Teffs to Tregs. Compared with mice exposed to BeO, mice treated with LPS showed a > 3-fold increase in the Teff/Treg ratio compared with the BeO-treated group (Figure 9D). Next, we examined the effector and Treg responses among HLA-DP2-CCL4/Be tetramer–binding CD4^+^ T cells. BeO-exposed HLA-DP2 Tg mice treated with LPS on day 14 had a significantly increased percentage of HLA-DP2-CCL4/Be tetramer–binding CD4^+^ Teffs in the lungs compared with BeO-exposed HLA-DP2 Tg mice (Figure 9E). Conversely, the frequency of HLA-DP2-CCL4/Be tetramer–binding CD4^+^ Tregs was greatly reduced in the LPS-treated group compared with BeO-exposed mice (Figure 9F). In addition, the Teff/Treg ratio of epitope-specific CD4^+^ T cells was significantly increased in HLA-DP2 Tg mice exposed to BeO plus LPS compared with the BeO-treated group (Figure 9G). Finally, mice treated with LPS showed a significant increase in the accumulation of neutrophils and increased protein in the BALF at day 15 compared with the BeO group (Figure 9, H and I), suggesting that additional environmental exposures have the capacity to modulate Be-induced adaptive immunity, lung inflammation, and injury.

## Discussion

Be exposure in the workplace results in activation of the innate immune response, characterized by the release of IL-1α and DAMPs that orchestrate the recruitment of inflammatory mononuclear cells and T and B cells (1, 4, 11, 34). In genetically susceptible individuals expressing βGlu69-containing *HLA-DPB1* alleles, an adaptive immune response ensues if a threshold quantity of Be is inhaled (1, 35). In an HLA-DP2 Tg murine model of Be-induced inflammation, BeO exposure resulted in the accumulation of Be-responsive CD4^+^ T cells in the lung and mononuclear infiltrates in a peribronchovascular distribution (3). In addition, BeO exposure expanded Tregs in the lungs, and Treg depletion caused increased accumulation of mononuclear infiltrates (3). Here, we demonstrate that Be exposure results in the accumulation of 3 distinct CD4^+^ T cell subsets in the lung parenchyma and that the amount of Be directly regulates the number of total and antigen-specific CD4^+^ T cells and Tregs in HLA-DP2 Tg mice. Depletion of Tregs in BeO-exposed mice skewed the T cell response toward Teffs, resulting in enhanced inflammation with increased neutrophil accumulation and MPO in the lungs. Conversely, Treg expansion with IL-2/αIL-2 complexes in BeO-exposed HLA-DP2 Tg mice suppressed the accumulation of pathogenic CD4^+^ T cells in the lung. Additionally, LPS exposure in the setting of BeO sensitization/boost resulted in a more proinflammatory lung microenvironment and induced the accumulation of large numbers of HLA-DP2-CCL4/Be–specific CD4^+^ Teffs. Thus, the current study defines the kinetics of Teff and Treg cell accumulation in the lung in response to BeO exposure and suggests the ability of expanded Tregs to modulate Be-induced adaptive immunity.

CD4^+^ Tregs are crucial players in maintaining homeostasis and controlling inflammation in both sterile and pathogenic settings (22, 36–40). CD4^+^ T cells recruited to the lungs of mice exposed to silica or Be complexes are composed of mixed populations of Th1 cells and Tregs (3, 41, 42). However, the phenotype and function of lung-resident CD4^+^ T cells and Tregs are not well characterized in Be-induced disease. CD44, CD103, and CD69 are expressed by tissue-resident T cells, and coexpression of CD103 and CD69 confirms the memory phenotype of lung-resident Tregs (43). Increased expression of CD103 and CD69 also distinguished lung-resident Tregs from peripheral Tregs that display low expression of these molecules. Depending on the amount of BeO administered, the frequency and number of total CD4^+^ T cells and Tregs changed over time. We observed a dose-dependent increase in the accumulation of total CD4^+^ T cells and Tregs comprising a bona fide tissue-resident CD103^+^CD69^+^ memory population compared with the recently emigrated CD103^–^CD69^+^ Teffs. However, the majority of Tregs coexpress CD103 and CD69, suggesting that these cells constitute the majority of tissue RM CD4^+^ T cells that control Be-induced inflammation, which is driven by pathogenic Th1-polarized Teffs. CD103^+^CD69^+^ tissue RM cells were also suppressive in a murine model of aspergillosis, while CD103^–^CD69^+^ resident cells were associated with a proinflammatory phenotype and induction of lung fibrosis (43).

CD4^+^ Tregs express a variety of molecules and transcription factors besides FoxP3 that define their functional status. In this study, we examined the expression of ICOS that is involved in the activation, differentiation, and function of Tregs, along with CTLA-4, GITR, and Nrp-1. ICOS is expressed on multiple CD4^+^ T cell subsets and controls various facets of T cell function and survival (44). ICOS is highly enriched in Tregs, and increased expression of ICOS is associated with enhanced proliferation and suppressive ability, suggesting a role in the maintenance of immune homeostasis (45). Also, in murine models of colitis and allergy-induced inflammation, increased expression of ICOS and CTLA-4 on Tregs was associated with immune suppression (46). The expression of GITR is associated with Treg activation (47) and controlling the expansion of Tregs in autoimmunity (48). Nrp, on the other hand, defines naturally occurring Tregs; however, it can also be upregulated on peripherally derived tissue-resident T cells (49, 50). Altogether, the expression of these molecules categorized Tregs into activated, effector, and memory phenotypes (47). Here, we found that a single BeO exposure induced upregulation of ICOS, CTLA-4, and GITR on tissue-resident Tregs independently of time as compared with splenic Tregs, with only a slight increase in the expression of Nrp-1 on lung-resident Tregs. Tregs migrate to the lung in response to chemokine receptor–ligand interactions. In the BALF of BeO-exposed HLA-DP2 Tg mice, we observed elevated levels of CXCL10, CCL19, and CCL21. CXCL10-dependent migration requires CXCR3 expression on T cells, while CCL19 and CCL21 direct T cell migration through the expression of CCR7 (23, 24). Our findings suggest that CXCL10, CCL19, and CCL21 play roles in the recruitment of T cells to the lung in response to Be-induced inflammation. Collectively, our findings suggest that, upon arrival in the lungs, Tregs express an activated phenotype.

We recently demonstrated that repeated BeO exposure of HLA-DP2 Tg mice resulted in the accumulation of lung CD4^+^ T cells specific for the HLA-DP2-CCL/Be epitope (13). Our analysis of antigen-specific T cells showed a dose- and time-dependent increase in the frequency and number of antigen-specific Tregs. However, multiple exposures concurrently increased the number of tissue-specific CD44^+^CD4^+^ Teffs. In this regard, our analysis of the Teff/Treg ratio suggests that the outcome of Be-induced inflammation and pathogenicity of antigen-specific CD4^+^ T cells depends on the Teff/Treg ratio. Twelve days after BeO sensitization, a significant increase in the Teff/Treg ratio was seen compared with the mice exposed to a single BeO exposure. Conversely, the ratio of Teff/Treg significantly decreased after boosting with BeO and analysis at day 21. The decrease in the Teff/Treg ratio in BeO sensitized/boosted mice suggests that boosting shifts the balance from RM CD4^+^ Teffs to tolerogenic antigen-specific CD4^+^ RM Tregs. Our ELISPOT data confirm the functional consequences of this transition with a significant reduction in the number of IFN-γ–secreting T cells after boosting at day 21 compared with sensitization only at day 12. This decrease in the Teff/Treg ratio and fewer IFN-γ–producing T cells in BeO sensitized/boosted HLA-DP2 Tg mice indirectly supports the suppressive ability of expanded tissue-resident Tregs in the lung, and this ability is driven by the quantity of antigen exposure in the lung microenvironment.

Treg induction and depletion studies in the current study confirm the protective role of BeO-induced CD4^+^ Tregs. Previous studies showed a regulatory role for Tregs in various autoimmune and inflammatory diseases (32). In humans, a mutation in the FoxP3 gene resulted in immune dysregulation polyendocrinopathy enteropathy X-linked (IPEX) syndrome (51, 52). Considering the importance of Tregs in our model of Be-induced disease, we found that depletion of Tregs using FoxP3-DTR Tg mice or anti-CD25 mAb increased the number of total effector and resident CD4^+^ T cells compared with the isotype-treated group or mice treated with IL-2/αIL-2 complexes. The CD25^hi^ targeting immunotoxin (denileukin diftitox) depletes Tregs by promoting cell death, resulting in strong CD8 responses that help in clearing cancer (53). In our studies, the use of anti-CD25 mAb (PC61) induced a similar response as that observed with denileukin diftitox in human studies. The total number of Tregs was not different in the treated groups (data not shown), and this was likely due to an overall increase in the total number of CD4^+^ T cells in the lungs of mice depleted of Tregs. The presence of an increased population of CD4^+^ Teffs was likely responsible for the enhanced inflammation-induced lung injury. Our findings in Be-induced disease are in close agreement with the role of Tregs in silicosis and other sterile particles, where exposure to silica particles resulted in the accumulation of leukocytes in the airways, but the loss of Tregs attenuated silica-induced fibrosis and enhanced the Th1 response (41). Furthermore, Tregs purified from BeO-exposed mice suppressed the proliferation of anti-CD3–stimulated CD4^+^ T cells in vitro in a similar manner to that observed in LPS-induced lung injury (32).

The bacterial toxin LPS induces strong inflammatory responses; however, the inclusion of LPS during the BeO sensitization phase has no effect on the number of HLA-DP2-CCL/Be–specific CD4^+^ T cells (data not shown). Conversely, when LPS was administered on day 14, it significantly increased the number of HLA-DP2-CCL/Be–specific CD4^+^ T cells in the lungs of BeO-exposed HLA-DP2 Tg mice (13). In the current study, we extended those findings to analyze total CD4^+^ T cells, Teffs, and Tregs, including antigen-specific T cell responses. Although the number of Tregs in the lung increased slightly after LPS exposure, the substantial increase in the number of Teffs resulted in a significantly increased Teff/Treg ratio. Similar to the total CD4^+^ T cell response, we observed an increase in the number of HLA-DP2-CCL4/Be–specific Teffs and an increased HLA-DP2-CCL4/Be–specific Teff/Treg ratio. LPS exposure also increased neutrophil accumulation in the lungs and increased protein leak, suggesting an enhanced inflammatory response.

CBD is characterized by persistent lung inflammation due to the inability of the human body to eliminate Be (35). As a result, once immunosuppressive treatment has begun, it is typically required for the life of the individual (54). Treatment is usually focused on prednisone with or without steroid-sparing agents such as methotrexate and azathioprine (54). However, these therapies are associated with side effects, and treatment alternatives are needed in both CBD and sarcoidosis. In this regard, studies suggest that Tregs are deficient in the lungs of subjects with CBD (31) and sarcoidosis (55, 56) or display impaired Treg function (56). Our findings of the ability of IL-2/αIL-2 complexes to modulate the number of Tregs in the lung and decrease the number of HLA-DP2-CCL/Be–specific CD4^+^ T cells, as well as the number of IFN-γ–producing T cells, in the lungs raise the possibility that modulation of Tregs may be a viable therapeutic alternative in granulomatous lung disease. These IL-2/αIL-2 complexes, along with high-dose IL-2, are currently being studied in oncology and autoimmunity.

Overall, this study provides a detailed characterization of tissue-resident CD4^+^ Teffs and Tregs in Be-induced disease. Be sensitization was characterized by an expansion of IFN-γ–producing Teffs, while the time period following the Be boost was dominated by an expansion of suppressive Tregs, including those specific for the HLA-DP2-CCL/Be complex. These findings are relevant to diseases characterized by repeated and persistent exposures and show the ability of additional environmental insults to modulate the underlying adaptive immune response in the lung.

## Methods

### Mice and treatment.

Six- to 8-week-old HLA-DP2 Tg FVB/N or C57BL/6 (B6) mice and HLA-DP2-FoxP3-DTR Tg B6 mice were exposed to 50 μL of either sterile PBS or PBS-containing 100 μg BeO (NIST, standard reference material 1877) via oropharyngeal aspiration as previously described (3, 18). HLA-DP2-FoxP3-DTR Tg mice were generated by crossing the in-house HLA-DP2 B6 mice with FoxP3-DTR Tg mice purchased from The Jackson Laboratory. Mice were lightly anesthetized with isoflurane to allow inhalation of sterile PBS or BeO, and a limulus amebocyte assay (Sigma-Aldrich) was performed to confirm that preparations contained < 20 μg of endotoxin. For short-term experiments, mice were sensitized at day 0 or days 0, 1, and 2 and euthanized at day 12. After sensitization, a separate cohort of mice were boosted with either BeO or PBS via oropharyngeal aspiration on days 14, 15, 18, and 19, and they were euthanized on day 21. In select experiments, mice were injected i.p. with either DT (1 μg/mouse), an anti–mouse CD25 antibody (BioXcell, PC61), or a murine IgG1 isotype control antibody (BioXcell, HRPN), as described previously (3). For enhancing the frequency of Tregs, mice were injected i.p. with anti–IL-2 (Invitrogen, JES-1A12)/αIL-2 complex mAb (Invitrogen) on days –1, 0, 1, and 7. In ICOS or CTLA-4 blocking experiments, HLA-DP2 Tg mice were injected with 300 μg of an anti–CTLA-4 antibody (BioXcell; clone UC10-4F10-11) or an anti-ICOS antibody (BioXcell; clone 7E.17G9) on day –1. Mice were maintained and bred in our animal facility.

### Preparation of single-cell suspensions of splenocytes and lung cells.

CD45-APC-Cy7 (10 μg/mouse) mAb (BioLegend, 30-F11) was injected i.v. via a retro-orbital route to discriminate tissue cells from circulating cells (29). Mice were euthanized under anesthesia, and lungs were perfused with ice-cold PBS. Spleens were pressed through a 100 μM cell strainer, and the strainer was rinsed with PBS. Lungs were removed, minced, and digested in complete culture media (RPMI-10), consisting of RPMI 1640 (HyClone) supplemented with 10% heat-inactivated FBS (HyClone) and penicillin and streptomycin (Invitrogen) and containing 1 mg/mL collagenase (Sigma-Aldrich). After 30 minutes, collagenase-digested lungs were sequentially disrupted with 16G and 18G needles. Collagenase was quenched with cold PBS, and lung cells were centrifuged at 250*g* at 4°C for 10 minutes. Lung cells were filtered through a 70 μM cell strainer and resuspended in RPMI-10. Spleens were processed through a 100 μM cell strainer and rinsed with PBS. Cells were pelleted by centrifugation at 250*g* at 4°C for 10 minutes. After ammonium chloride lysis of erythrocytes, splenocytes were filtered through a 70 μM cell strainer and resuspended in RPMI-10.

### Preparation of lung homogenates, isolation of BALF, and chemokine ELISA.

Snap-frozen lung tissue was thawed in 0.5 mL PBS containing 0.05% Tween 20 and protease inhibitors (MilliporeSigma) and homogenized (Omni International) at 35,000 rpm for 30 seconds on ice. Homogenates were centrifuged at 10,000*g* for 10 minutes at 4^o^C. Supernatants were transferred to a 96-well tissue culture plate and stored at –80°C. Supernatants were thawed and treated with nuclease (MilliporeSigma) (Benzonase; 25 units for 30 minutes at 37°C) and centrifuged at 400*g* for 5 minutes through a filter plate (MilliporeSigma, multiscreen plate). BALF was collected in 1 mL of PBS by tracheal cannulation, as previously described (3, 12). BALF was centrifuged at 2000*g* for 5 minutes at 4°C, and the acellular fraction was frozen at –80°C for analysis of lung injury. CXCL10 (IP-10) in lung lysate and CCL19 and CCL21 in the BALF were measured using R&D DuoSet ELISA kits. In total, 50 μL of lung lysate or BALF was assayed.

### Flow cytometry.

Fluorescently labeled antibodies to cell surface antigens were diluted in PBS containing 1% FCS, 0.05% sodium azide, and 0.5 μg/mL CD16/CD32 (Tonbo; 2.4G2). Cells were incubated for 30 minutes at 4°C, washed, and resuspended in FACS buffer. The following antibodies were used for multi-parameter FACS analysis: CD3 (Tonbo; 17A2), CD44 (Tonbo; IM7), GITR (eBioscience; DTA-1); CD4 (RM4-5, RM4-4), CD8 (53-6.7), CD69 (H1.2F3), B220 (RA3-682), CD103 (2E7), Nrp-1(3E12), ICOS (C398.4A), and CTLA-4 (UC10-4B9) were all purchased from BioLegend, unless specified otherwise. To remove innate cells in our analysis of tissue-resident CD4^+^ T cells and tetramer analysis, antibodies directed against CD11c (N418), CD11b (M1/70), IA/IE (M5.114.15.2), HLA-DP (purified from hybridoma, B7.21), and Ly6G (1A8) were purchased from eBioscience. For HLA-DP2-CCL4/Be tetramer staining, lung cells were stained as previously described (13, 17). Intracellular staining for FoxP3 was performed according to the manufacturer’s protocol (Thermo Fisher Scientific). Data were obtained using a BD FACS Canto and Celesta Flow Cytometers and analyzed using FlowJo software (v9.9.6; Treestar, Inc.).

### Analysis of IFN-γ by ELISPOT assay.

ELISPOT plates (ImmunoSpot M200, BD Biosciences) were coated with IFN-γ capture mAb (eBioscience; AN18) overnight and blocked with RPMI-10 for 2 hours at room temperature (3). Lung CD4^+^ T cells were positively selected using magnetic beads, and 2.5 × 10^4^ CD4^+^ T cells were combined with 5 × 10^5^ irradiated spleen cells in triplicate from an unexposed HLA-DP2 Tg mouse. Cells were incubated overnight at 37°C with medium or 100 μM BeSO_4_. IFN-γ detection mAbs (eBioscience; R4-6A2) were added, and spots were visualized by avidin-horseradish peroxidase and 3-amino-9-ethylcarbazole substrate reagent (BD Biosciences). ELISPOT plates were analyzed using a CTL Immunospot Analyzer (Cellular Technology Ltd), and results are reported as mean ± SEM spot-forming units (SFUs) per well minus background SFUs (Media alone).

### Lung injury endpoints and MPO ELISA.

To quantitate protein, BALF was assayed in a BCA Protein Assay (Thermo Fisher Scientific). Total protein was determined using a BSA standard curve according to the manufacturer’s instructions (MilliporeSigma). Absorbance readings were measured at 620 nm using a VMax microplate reader (Molecular Devices). Concentrations were calculated using Prism (v.5.0d). To assess MPO levels, a double sandwich ELISA was performed according to manufacturer’s protocol (R&D). Coating buffer, assay diluent, SA-HRP, and TMB substrate were from eBioscience. Absorbance was measured at 450 nM, and absorbance readings were obtained using a VMax microplate reader.

### Histology.

Lungs were inflated, stored in 10% neutral-buffered formalin for 24 hours, and transferred to 70% ethanol for histopathological analysis. H&E staining was done by the Histowiz. To quantitate mononuclear cell infiltrates, whole slide imaging was performed on H&E-stained lung sections cut from formalin-fixed paraffin-embedded tissue. Pyramidal tiff files were analyzed using QuPath software (v.0.2.3). Briefly, stain vector values were automatically determined, and cells were counted by adjusting the cell detection threshold to maximize the difference between areas containing perivascular mononuclear infiltrates and unaffected areas (13).

### In vitro Treg suppression and transwell assay.

CD4^+^ T cells were purified from lungs and spleen of BeO-exposed and spleen of PBS-treated mice at day 21. Tconv cells were purified from naive spleen. Teffs (CD44^hi^CD25^lo^) and Tregs (CD25^+^) were sorted from purified CD4^+^ T cells on FACS ARIA II fusion (BD Biosciences). Tconv cells were stained with 1 μM carboxy fluorescein isothiocyanate (CFSE; Invitrogen) and cocultured with Teffs or Tregs in a 1:1 ratio in a 96-well anti-CD3–coated (1–2 μg/mL, clone 145-2C11, Thermo Fisher Scientific) plates for 5 days. Transwell assays were performed in a 96-well transwell plate (Corning). Loss of CFSE was examined by flow cytometry at day 5.

### Statistics.

A 1-way ANOVA, Mann-Whitney *U* test, and 2-tailed unpaired Student’s *t* test were used to determine significance of differences between groups (Prism v9.2.0, GraphPad Software.). *P* < 0.05 was considered statistically significant.

### Study approval.

All experiments were approved by the IACUC of the University of Colorado Anschutz Medical Campus, in accordance with *Guide for the Care and Use of Laboratory Animals* (National Academies Press, 2011). The University of Colorado Anschutz Medical Campus is accredited by the American Association for Accreditation of Laboratory Animal Care.

## Author contributions

SMA, DGM, and APF designed research; SMA, DGM, AKM, and APF performed research; SMA, DGM, and APF analyzed data; and SMA, DGM, and APF wrote the manuscript.

## Supplementary Material

Supplemental data

## Version 1 07/12/2022

In-Press Preview

## Version 2 08/22/2022

Electronic publication
