# Supplementary material for: Protective role of tissue-resident Tregs in a murine model of beryllium-induced disease
Source: JCI Insight. 2022 Aug 22;7(16):e156098. doi: 10.1172/jci.insight.156098 (PMC9462505; doi:10.1172/jci.insight.156098)
Supplement: Supplemental data [file jciinsight-7-156098-s130.pdf]

Supplementary Figure 1

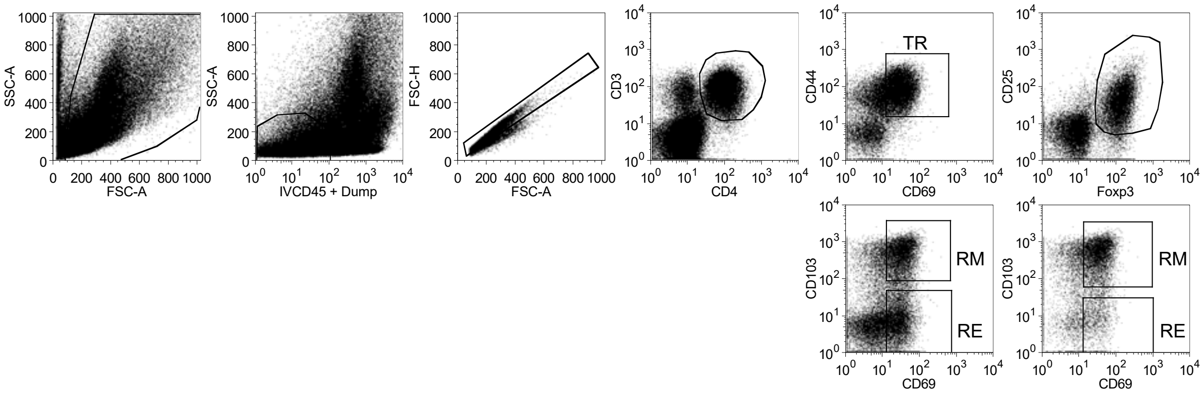

Supplemental Figure 1. Gating strategy for the identification of tissue-resident CD4$^+$ T cells. Density plots show gating strategy for the identification of tissue-resident CD4$^+$ T cells present in the CD45 negative fraction of the lungs of HLA-DP2 Tg mice sensitized and boosted with BeO (100 μg) on days 0, 1, and 2, 14, 15, 18 and 19 and examined at day 21. TR denotes tissue-resident T cells while RM and RE denote resident memory and resident effector T cells, respectively.

Supplementary Figure 2

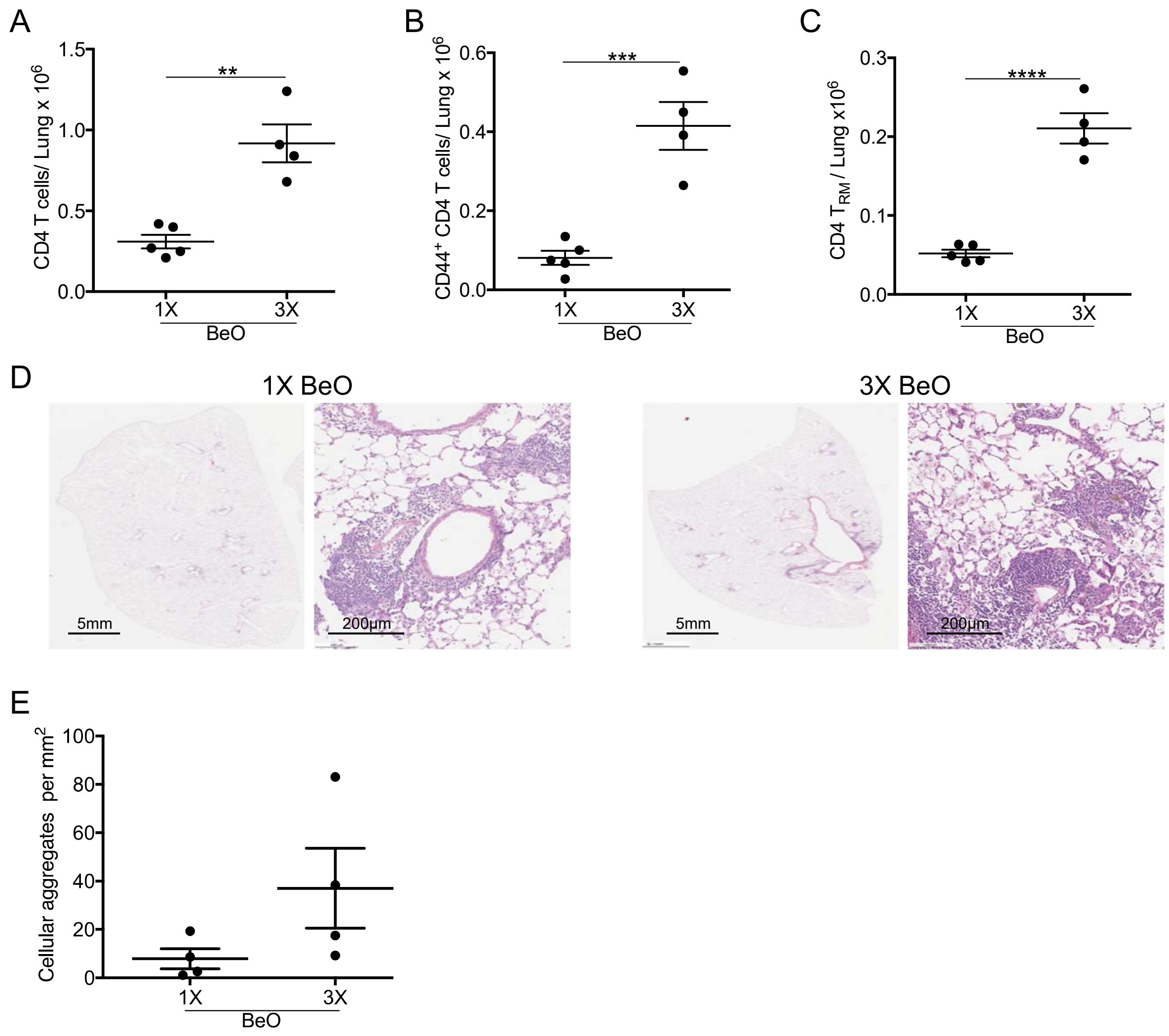

Supplemental Figure 2. BeO sensitization induces an increased accumulation of tissue-resident CD4$^+$ T cells in the lungs of HLA-DP2 Tg mice on day 12. (**A-C**) The number of total CD4$^+$ T cells (**A**), CD44$^+$ effector T cells (**B**), and CD103$^+$CD69$^+$ resident memory CD4$^+$ T cells (**C**) in the lungs at day 12. (**D**) Representative H&E staining of lung sections of HLA-DP2 Tg mice treated with 1 (1X, left) and 3 (3X, right) doses of BeO (100 µg) are shown at low and high magnification. A scale bar is shown in the lower left of each image. (**E**) Quantification of cellular aggregates in the lungs of HLA-DP2 Tg mice exposed to one (1X) or three (3X) doses of BeO. Data (mean ± SEM) are representative of three independent experiments (3-5 animals per group). Student's t test was used to test for differences. $^{**}p < 0.01$, $^{***}p < 0.001$, $^{****}p < 0.0001$.

Supplementary Figure 3

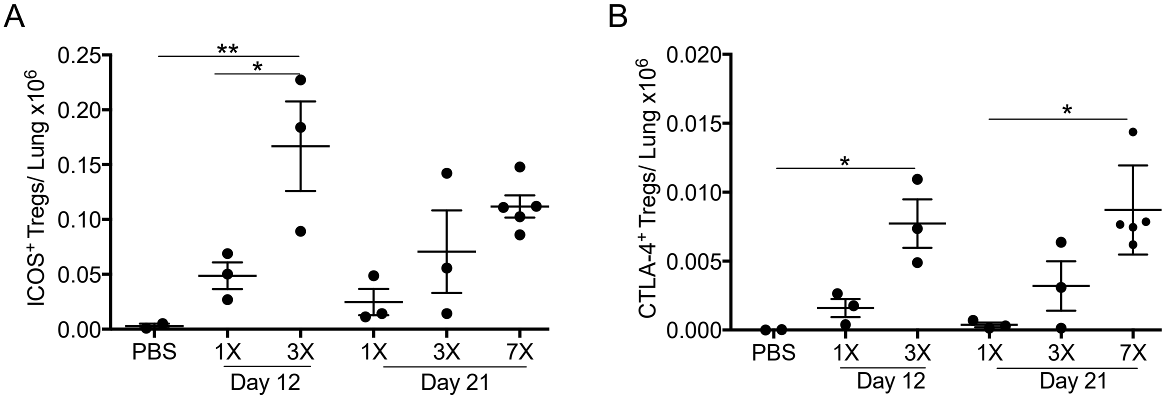

Supplemental Figure 3. Increased expression of ICOS and CTLA-4 on tissue-resident Tregs in the lungs of BeO-exposed HLA-DP2 Tg mice. (**A-B**) Number of ICOS (**A**) and CTLA-4 (**B**) expressing tissue-resident $CD25^+FoxP3^+$ regulatory $CD4^+$ Tregs in the lungs of HLA-DP2 Tg mice exposed to one (1X), three (3X), and seven (7X) doses of BeO and analyzed at day 21. Data (mean ± SEM) are representative of three independent experiments. One-way ANOVA was used to test for differences. *$p < 0.05$, **$p < 0.01$, ***$p < 0.001$, ****$p < 0.0001$.

Supplementary Figure 4

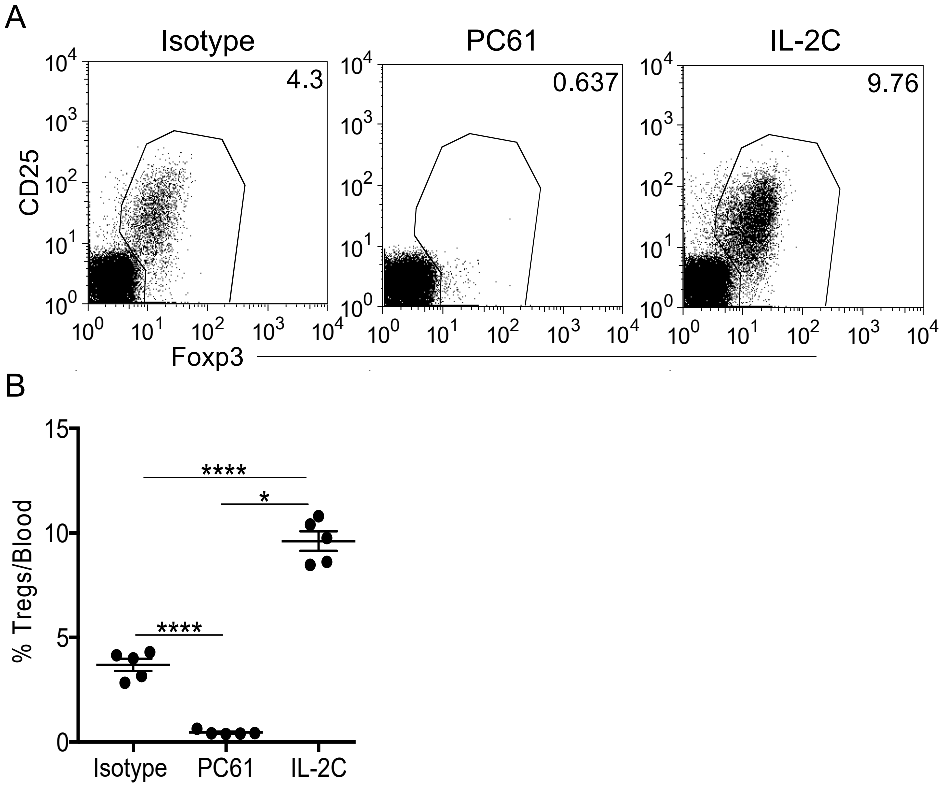

Supplemental Figure 4. Expression of CD4⁺ FoxP3⁺ T cells in the blood of BeO-exposed mice after antibody treatment. (A) Representative flow cytometric dot plots of $CD4^+$ $CD25^+FoxP3^+$ Tregs in blood on day 8 after HLA-DP2 Tg mice were sensitized to BeO on days 0, 1 and 2 and treated with either isotype control antibody, anti-CD25 mAb (PC61), or IL2/αIL-2 complexes (IL-2C, IL-2/anti-IL2(JES6-1)). (**B**) Cumulative frequency of $CD4^+$ Tregs in the blood at day 8. Data (mean ± SEM) are representative of two independent experiments. One-way ANOVA was used to test for differences. $*p < 0.05$, $**p < 0.01$, $***p < 0.001$, $****p < 0.0001$.

Supplementary Figure 5

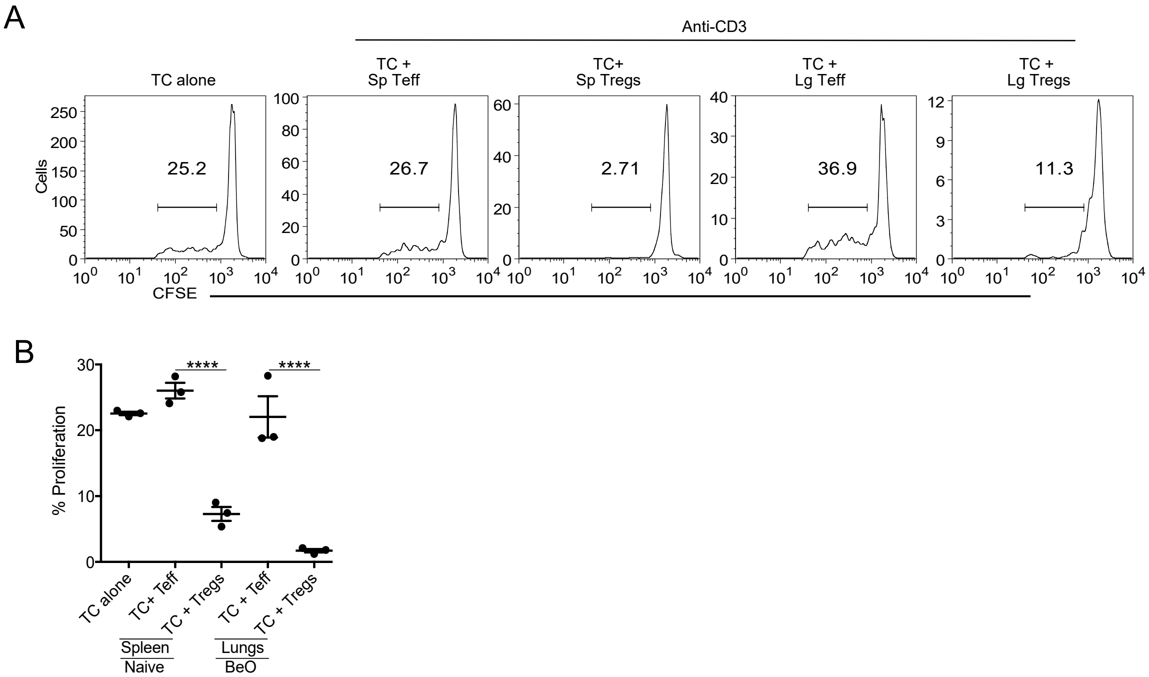

Supplemental Figure 5. Be-experienced Tregs suppress in vitro proliferation of anti-CD3 activated CD4$^+$ T cells in a contact dependent fashion. (**A**) Representative histograms show proliferation of CFSE-labeled naive CD4$^+$ T cells (TC) stimulated with anti-CD3 (1 µg/ml) and cultured in vitro in a 1:1 ratio with spleen (Sp) or lung (Lg)-resident effector (CD44$^+$CD25$^-$) or regulatory (CD25$^+$FoxP3$^+$) CD4$^+$ T cells obtained on day 21 from BeO-sensitized/boosted HLA-DP2 Tg mice. Peripheral T effectors (CD44$^+$CD25$^-$) and T regs (CD25$^+$FoxP3$^+$) sorted from the spleen (Sp) of naive mice were used as control cells. (**B**) Cumulative frequency of proliferation of CFSE-labeled TC cells on day 5. Data (mean ± SEM) are representative of three independent experiments. One-way ANOVA was used to test for differences. *p < 0.05, **p < 0.01, ***p < 0.001, ****p < 0.0001.
